# Sociodemographic factors associated with patients hospitalised for coccidioidomycosis in California and Arizona, State Inpatient Database 2005–2011

**DOI:** 10.1017/S0950268820002836

**Published:** 2020-11-20

**Authors:** D. Kupferwasser, L. G. Miller

**Affiliations:** 1 Division of Infectious Diseases Lundquist Institute for Biomedical Innovation at Harbor-UCLA Medical Center, USA; 2 Claremont Graduate University, Claremont, California, USA; 3 Division of Infectious Diseases Harbor-UCLA Medical Center, USA; 4 David Geffen School of Medicine at UCLA, USA

**Keywords:** Epidemiology, infectious disease epidemiology, public health

## Abstract

Coccidioidomycosis is endemic in the Southwestern United States. Disseminated infection can be life-threatening and is responsible for hospitalisation and significant healthcare resource utilisation. There are limited data evaluating factors associated with hospitalisation for coccidioidomycosis. We conducted a cross-sectional study to assess incidence and factors associated with coccidioidomycosis-associated hospitalisation in California and Arizona. We analysed hospital discharge data obtained from the State Inpatient Dataset for California and Arizona between 2005 and 2011 and performed multivariable logistic regression examining factors associated with coccidioidomycosis-associated hospitalisation. During our time frame, we found 23 758 coccidioidomycosis-associated hospitalisations. Coccidioidomycosis incidence was over sixfold higher in Arizona compared to California (198.9 *vs.* 29.6/100 000 person-years). In our multivariable model, coccidioidomycosis-associated hospitalisation was associated with age group 40–49 years (referent group: age 18–29 years, adjusted odds ratio (aOR) = 1.50 (95% confidence interval (CI) 1.43–1.59)), African American race (referent group: Caucasian, aOR = 1.98 (95% CI 1.89–2.06)), residing in a large rural town (referent group: urban area, aOR = 2.28 (95% CI 2.19–2.39)), uncomplicated diabetes (aOR = 1.47 (95% CI 1.41–1.52)) chronic obstructive pulmonary disease (aOR = 1.59 (95% CI 1.54–1.65)) and higher number of comorbidities (aOR = 1.02 (95% CI 1.02–1.03) for each point in the Elixhauser score). Identifying persons at highest risk for hospitalisation with coccidioidomycosis may be helpful for future prevention efforts.

## Introduction

Coccidioidomycosis is a fungal infection caused by the inhalation of *Coccidioides* species spores. This infection is most frequently found in areas where the soil is dry and alkaline, including the southwestern United States, parts of Mexico and Central and South America [1, 2]. The majority of infections are asymptomatic with symptomatic infections presenting as flu-like illness that is often self-limiting [3]. However, an estimated 3–5% of symptomatic coccidioidomycosis infections disseminate, and one-third of these cases are fatal [3]. Additionally, in a small proportion of coccidioidomycosis patients may require prolonged suppressive therapy following initial treatment course to prevent relapse [3]. Recently, concern has grown over a reported increase in the risk of exposure to *Coccidoides spp*. spores for populations in endemic areas, specifically military personnel, prisoners and solar panel workers [4–7].

In the U.S., coccidioidomycosis is a nationally notifiable disease. Data from the Centers for Disease Control and Prevention's National Notifiable Disease Surveillance System (NNDSS) have demonstrated secular shifts in coccidioidomycosis incidence. For example, coccidioidomycosis incidence has increased from 5.3/100 000 population in 1998 to 42.6/100 000 population in 2011 [8]. The factors that are responsible for this alarming increase in nationally reported *Coccidioidomycosis* cases are poorly understood. However, climate change, and increased migration of susceptible person to endemic areas have been suggested as possible explanations for this increase [9]. Of note, coccidioidomycosis is a reportable disease in California and Arizona, suggesting that the incidence increase may not be attributed to underreporting [10].

Coccidioidomycosis represents a substantial healthcare burden within California and Arizona, two highly endemic states where greater than 95% of all cases are reported [11–13]. Medical cost for the treatment of coccidioidomycosis is high; in Arizona, the cost for hospitalisations due to coccidioidomycosis was estimated to be $86 million in 2007 [14, 15]. Compounding the healthcare burden of coccidioidomycosis is the difficulty in diagnosing coccidioidomycosis which is commonly misdiagnosed for bacterial community-acquired pneumonia [16]. Misdiagnoses can contribute to an increase in the cost for care due to prolonged hospital stay [16]. Antifungal treatment costs range from $5000 to 20 000 annually with critically ill patients accruing additional costs due to intensive care support over many days in the hospital [17]. Additionally, antifungal treatments are also not without adverse effects, such as hepatotoxicity, gastrointestinal discomfort and neurologic side effects such as headache [18].

While there are data examining risk factors for coccidioidomycosis, there are limited studies evaluating factors associated with coccidioidomycosis hospitalisation. Moreover, several of these studies utilised national administrative data sources that do not hone down on the U.S. states that are most affected by coccidioidomycosis [19–21]. Therefore, we performed an investigation examining inpatient incidence of hospitalisation for coccidioidomycosis using state-level data from large highly endemic areas.

## Materials and methods

We conducted a cross-sectional study to assess the sociodemographic and comorbidity factors associated with coccidioidomycosis-associated hospitalisation *vs.* hospitalisation associated with other causes in California and Arizona. Hospital discharge data were obtained from the State Inpatient Dataset (SID) for both California and Arizona for years 2005–2011. The SID represents all-payer inpatient discharge information collected from nonfederal (e.g. not military, Veterans Administration or Indian Health Services) hospitals. Combining both California and Arizona SID for years 2005–2011 resulted in a cohort of over 33.0 million inpatient stays.

The study population included all inpatient discharges for The Healthcare Cost and Utilization Project (HCUP)-defined hospitals from California and Arizona for the years 2005–2011. A coccidioidomycosis case was defined as a primary or secondary diagnosis identified by the International Statistical Classification of Diseases and Related Health Problems version 9 (ICD-9) codes assigned for coccidioidomycosis (Table 1). Population denominator data for the HCUP SID datasets consist of decennial census with annual estimates for years 2005–2007 and the American Community Survey for years 2007–2011 [22]. We calculated rates of coccidioidomycosis-associated hospitalisation by race and ethnicity for the years 2005–2011 using data from the 2010 U.S. Census, assuming a stable population for our study time frame.

**Table 1.** Demographic features and comorbidities for coccidioidomycosis-associated hospitalisation patients from the California and Arizona State Inpatient Datasets, 2005–2011

	*N* (%)	
Total	23 758 (0.1)	33 622 215 (99.9)
Characteristic	Coccidioidomycosis (case)	(Control)
Sex		
Female	8687 (37.1)	19 080 000 (58.6)
Male	14 735 (62.9)	14 735 (41.4)
Age (years)		
0–17	1025 (4.3)	6 729 194 (20.0)
18–29	2888 (12.2)	4 125 162 (12.5)
30–39	3566 (15.1)	3 441 015 (10.4)
40–49	4544 (19.2)	3 184 698 (9.6)
50–59	4393 (18.6)	3 712 402 (11.2)
>60	7251 (30.6)	1 210 317 (36.3)
Race/ethnicity		
White	12 047 (54.5)	1 810 474 (54.3)
African American	2681 (12.1)	2 266 488 (7.3)
Hispanic	5792 (26.2)	9 041 211 (29.2)
Native American/Alaska native	905 (4.1)	2 040 092 (6.6)
Asian/Pacific Islander	425 (1.9)	213 243 (0.7)
Others	254 (1.2)	594 988 (1.9)
Rural–Urban patient location		
Urban	17 985 (84.2)	30 966 060 (92.1)
Large rural town	2539 (11.9)	1 601 255 (5.0)
Small rural town	595 (2.8)	634 359 (2.0)
Isolated rural	222 (1.1)	290 338 (0.9)
Household income*		
First quartile	6777 (30.4)	9 074 550 (28.0)
Second quartile	5486 (24.6)	8 335 102 (25.7)
Third quartile	5341 (24.0)	8 068 555 (25.0)
Fourth quartile	4668 (21.0)	6 927 733 (21.3)
Mortality		
Died in hospital	589 (2.5)	639 629 (1.9)
Alive at discharge	23 147 (97.5)	327 283 392 (98.1)
State		
CA	10 970 (46.3)	21 196 962 (63.3)
AZ	12 700 (53.7)	12 449 011 (36.7)
Comorbidity		
AIDS		
Yes	60 (0.3)	56 682 (0.2)
No	23 698 (99.7)	33 589 291 (99.8)
Diabetes (uncomplicated)		
Yes	4678 (19.7)	4 097 289 (12.3)
No	19 080 (80.3)	29 586 682 (87.7)
Obesity		
Yes	1620 (6.8)	2 380 693 (7.1)
No	22 138 (93.2)	30 935 037 (92.9)
Chronic obstructive pulmonary disease		
Yes	4922 (20.7)	4 141 441 (12.4)
No	18 836 (79.3)	29 253 060 (87.6)

* The quartiles are identified by values of 1 to 4, indicating the poorest to wealthiest populations. First quartile represents 0-25th percentile, second quartile represents 26th-50th percentile, third quartile represents 51th-75th percentile, and the fourth quartile represents 76th -100th percentile.

Our primary outcome was coccidioidomycosis-associated hospitalisation. To assess factors that may be associated with these hospitalisations, we examined a pre-selected group of sociodemographic and patient-level comorbidity factors. These factors were chosen based on possible associations suggested by the medical literature to be associated with a higher rate of coccidioidomycosis infection or severe coccidioidomycosis infection [3, 19] and the fact that they were available in the SID databases. Additionally, we examined demographic factors that we believed may be important confounders.

We hypothesised rural persons were at a higher risk given the relative lack of pavement, more contact with soil and potentially more exposure to the outdoor environment. Urbanisation was stratified into four rural–urban patient locations, urban (metropolitan), large rural town (micropolitan), small rural town and isolated small rural area. The stratification used by HCUP to generate the condensed urbanisation variable within the SID database is based on rural–urban commuting areas (RUCA) and assignment into ZIP codes using population and commuting information from the 2000-year Census [23]. Patient household income was included in our analyses to identify the presence of potential health disparities within different socioeconomic groups for coccidioidomycosis-associated hospitalisation. We hypothesised persons of lower socioeconomic status were at a higher risk attributed to financial barriers to healthcare and overall lower health status compared to persons with higher socioeconomic status. Age was stratified into six categories, (0–17, 18–29, 30–39, 40–49, 50–59 and >60 years), similar to prior work that also used administrative level data [20]. The race was categorised as Caucasian (non-Hispanic), African American (non-Hispanic), Hispanic, Native American or Alaska Native (non-Hispanic), Asian/Pacific Islander (non-Hispanic) and other or multiple races (non-Hispanic). Household income is represented by the quartile of the median household income based on the patient's zip code and was included as a community level socioeconomic factor. The state where the hospital is located (California or Arizona) was also included as a variable in the analysis. Comorbidities were measured using the Elixhauser comorbidity index [24–26]. The Elixhauser Index, a validated scale of co-morbidity from administrative databases was calculated using the Elixhauser Comorbidity Software Version 3.7 [24].

Environmental factors such as seasonal rainfall, earthquakes and soil disruptions have been shown to be associated with higher incidences of reported coccidioidomycosis cases [27, 28]. We hypothesised that coccidioidomycosis cases would increase following periods of increased rainfall. The incubation for coccidioidomycosis is not well established, therefore we performed four models for the lag between rainfall and coccidioidomycosis-associated hospitalisation incidence (0, 1, 2 and 3 months) [27, 29–31]. The average monthly rainfall was calculated using data obtained from the National Oceanic and Atmospheric Administration [32]. Due to monthly rainfall variability between Arizona and California, such as Arizona's monsoon season beginning in June and continuing through September while California experiences its rainfall season between October and April, California and Arizona, the analysis was done separately for each state. Associations were measured using Spearman's correlation.

Our study population consisted of persons hospitalised due to coccidioidomycosis, cases and non-cases. Non-cases represent hospitalised patients admitted for non-coccidioidomycosis diagnoses. Bivariate analyses were performed and all factors with a *P* value of <0.20 in these analyses were included in a logistic multivariable regression analysis. To assess multicollinearity among variables in our multivariable model, variance decomposition proportion (VDP) analysis was performed using the SAS macro COLLIN [33]. All analyses were performed using SAS^®^ version 9.2 (SAS Institute Inc., Cary, North Carolina) [34].

## Results

We found a total of 33 622 215 patients were hospitalised in Arizona and California between the years 2005 and 2011. Among these, there were 23 758 hospitalisations with an associated diagnosis of coccidioidomycosis recorded in the dataset. Primary diagnosis of coccidioidomycosis accounted for 72% of cases and a secondary diagnosis of coccidioidomycosis accounted for 28% of cases. Arizona had an over sixfold higher coccidioidomycosis-associated hospitalisation incidence rate compared to California over the study time frame, 198.9/100 000 *vs.* 29.6/100 000 population (Table 2). The most common coccidioidomycosis diagnosis for both states was ‘primary coccidioidomycosis’ (13 919, 58.6%). In order of decreasing frequency, the next most common diagnoses were chronic and unspecified pulmonary, progressive coccidioidal not elsewhere classified, coccidioidal meningitis and primary cutaneous coccidioidomycosis and not elsewhere specified. although the relative ranking of these diagnoses varied by state (Table 2).

**Table 2.** Coccidioidomycosis diagnosis rates per 100 000 persons

		*N* (rate/100 000)	
Diagnosis codes of coccidioidomycosis	*N* (%)	CA	AZ
Hospitalisations	23 758 (100)	11 043 (29.6)	12 715 (198.9)
International classification of diseases codes (ICD-9)			
Primary coccidioidomycosis (114.0)		6107 (16.4)	7812 (122.2)
Primary cutaneous and NOS (114.1 and 114.9)		553 (1.5)	693 (10.8)
Coccidioidal meningitis (114.2)		1316 (3.5)	983 (15.4)
Progressive coccidioidal NEC (114.3)		1855 (5.0)	1455 (3.9)
Chronic and unspecified pulmonary (114.4 and 114.5)		1295 (3.5)	1863 (29.1)

**Fig. 1.**
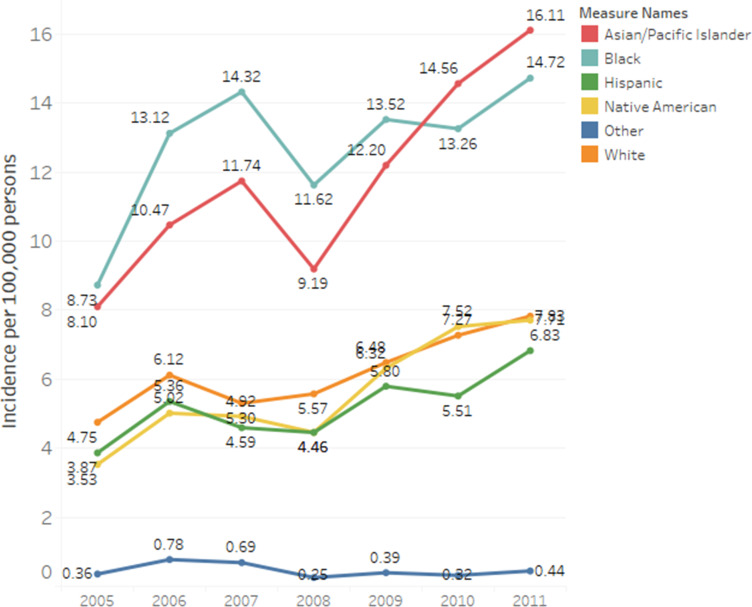
Annual coccidioidomycosis hospital incidence rates per 100 000 persons by racial and ethnic groups. Yearly coccidioidomycosis hospital incidence rates per 100 000 persons are presented for racial/ethnic groups: Asian/Pacific Islander, African American, Hispanic, Native American, Caucasian and others.

Annual incidence rates of coccidioidomycosis-associated hospitalisations differed by race/ethnicity (Fig. 1). Asian/Pacific Islanders and African Americans had the highest incidence rates across all years (Fig. 1). The average annual incidence of coccidioidomycosis-associated hospitalisation per 100 000 persons for Asian/Pacific Islanders and African Americans was 11.8/1 000 000 persons (95% CI 9.1–14.4) and 12.8/100 000 persons (95% CI 10.9–14.6), respectively. Average annual incidence among Caucasians was 6.2/100 000 persons (95% CI 5.2–7.2), 5.6/100 000 persons (95% CI 4.2–7.1) among Native American, 5.2/100 000 persons (95% CI 4.3–6.1) among Hispanics and 0.5/100 000 persons (95% CI 4.1–7.1) among other race/ethnicities.

**Fig. 2.**
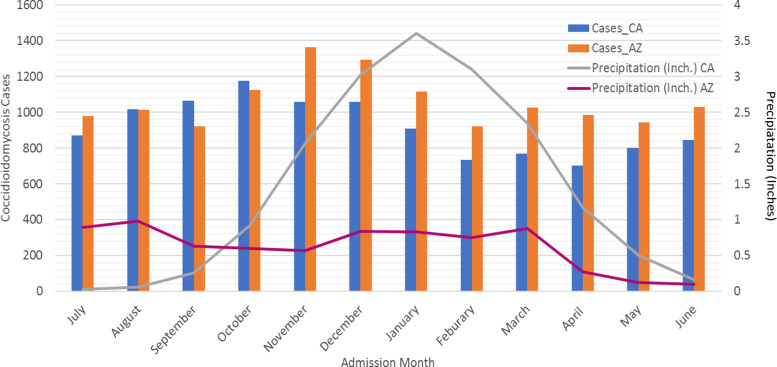
Monthly hospitalisation for Arizona and California with average seasonal precipitation amounts. Bar graph represents number of cases of coccidioidomycosis by month of hospital admission for both California and Arizona. Monthly precipitations in inches is shown as a line graph for both California and Arizona. Abbreviations: CA: California, AZ: Arizona, Inch: inches.

In Arizona, when examining the association of rainfall with coccidioidomycosis-associated hospitalisation, we found that monthly hospitalisations associated with coccidioidomycosis showed high-incidence periods during the winter months of October, November and December (Fig. 2). The average precipitation for these months ranged from 0.60 to 0.84 inches. September and February showed the lowest incidence of hospitalisations. In Arizona, peak precipitation during this time period occurred in August and March. We found no correlation between coccidioidomycosis-associated hospitalisations and all four lag times (0, 1, 2 and 3 months) (Table 3).

**Table 3.** Seasonal precipitation and coccidioidomycosis hospitalisation for California and Arizona

State	Lag time	Spearman's correlation coeff.	*P* value
California	No lag	−0.032	0.77
	1-month lag	−0.28	0.01
	2-month lag	−0.41	0.0001
	3-month lag	−0.42	0.0001
Arizona	No lag	−0.13	0.24
	1-month lag	−0.2	0.07
	2-month lag	−0.18	0.11
	3-month lag	0.1	0.38

In California, the incidence of coccidioidomycosis-associated hospitalisations peaked in October. Peak rainfall occurred in December, January and February (3.02, 3.60 and 3.10 inches, respectively). A significant negative correlation was found between seasonal rainfall and coccidioidomycosis-associated hospitalisations for California for the 1-, 2- and 3-month lag times but not for the zero month lag time (Table 3).

In bivariate analysis, we found numerous predictors associated with coccidioidomycosis-associated hospitalisations that were included in our multivariable analysis (Table 4). In multivariable analysis, among demographic factors, coccidioidomycosis-associated hospitalisation were inversely associated with female gender (adjusted odds ratio (aOR 0.40 (95% CI 0.40–0.41)). Persons in the age group (40–49) years were more likely to have coccidioidomycosis-associated hospitalisation compared to our referent group: age 18–29 years, aOR = 1.50 (95% CI 1.43–1.59)). Similarly, other non-elderly adult age categories were positively associated with coccidioidomycosis-associated hospitalisation: age 30–39 years: (aOR 1.42 (95% CI 1.35–1.50)); and age 50–59; (aOR 1.07 (95% CI 1.02–1.30)). However, children age 0–17 years: (aOR 0.17 (95% CI 0.16–0.18)) and adults age >60 (aOR 0.51 (95% CI 0.49–0.54)) years were less likely to have coccidioidomycosis-associated hospitalisation. In term of race, compared to Caucasians in our multivariable model, we found that coccidioidomycosis-associated hospitalisations were associated with African American race (aOR 1.98 (95% CI 1.89–2.06)), Hispanic ethnicity (aOR 1.32 (95% CI 1.27–1.36)), Native American/Alaska Native race (aOR 1.26. (95% CI 1.18–1.35)) and other race/ethnicity (aOR 1.07 (95% CI 0.94–1.22)).

**Table 4.** Predictors of coccidioidomycosis hospitalisation (SID CA & AZ, 2005–2011)

	Bivariate	Multivariable
Total	33 645 973	33 645 973
Characteristic	OR (95%) CI	aOR (95%) CI
**Sex**		
Female	0.41 (0.40–0.43)	0.40 (0.39–0.41)
Male	Ref.	Ref.
**Age (years)**		
0–17	0.22 (0.21–0.24)	0.17 (0.16–0.18)
18–29	Ref.	Ref.
30–39	1.48 (1.41–1.56)	1.42 (1.35–1.50)
40–49	1.92 (1.83–2.01)	1.50 (1.43–1.59)
50–59	1.70 (1.61–1.77)	1.07 (1.02–1.30)
>60	0.86 (0.83–0.90)	0.51 (0.49–0.54)
**Race/ethnicity**		
Caucasian	Ref.	Ref.
African American	1.66 (1.59–1.73)	1.98 (1.89–2.06)
Hispanic	0.90 (0.87–0.93)	1.32 (1.27–1.36)
Native American/Alaska native	0.62 (0.58–0.67)	1.26 (1.18–1.35)
Asian/Pacific Islander	2.80 (2.54–3.01)	0.90 (0.81–1.01)
Other	0.60 (0.53–0.68)	1.07 (0.94–1.22)
**Rural−urban patient location**		
Urban	Ref.	Ref.
Large rural town	2.43 (2.33–2.53)	2.28 (2.19–2.39)
Small rural town	1.48 (1.36- 1.60)	0.93 (0.85–1.01)
Isolated rural	1.16 (1.02 −1.33)	0.95 (0.84–0.91)
**Household income**		
First quartile	1.09 (1.06, 1.13)	0.87 (0.84–0.91)
Second quartile	0.97 (0.93, 1.01)	0.78 (0.75–0.81)
Third quartile	0.98 (0.94, 1.02)	0.87 (0.83–0.81)
Fourth quartile	Ref.	Ref.
**Mortality**		
Died in Hospital	1.34 (1.24–1.45)	1.12 (1.03–1.22)
Alive at discharge	Ref.	Ref.
**Elixhauser Index**		
Score	1.03 (1.03–1.03)	1.02 (1.02–1.03)
**State**		
CA	Ref.	Ref.
AZ	5.90 (5.74–6.01)	6.42 (6.25–6.60)
**Comorbidity**		
AIDS		
Yes	1.5 (1.16–1.90)	0.75 (0.58–0.99)
No	Ref.	Ref.
Diabetes (uncomplicated)		
Yes	1.75 (1.7–1.80)	1.47 (1.41–1.52)
No	Ref.	Ref.
Obesity		
Yes	0.95 (0.90–1.00)	0.78 (0.74–0.83)
No	Ref.	
Chronic pulmonary disease		
Yes	1.84 (1.79–1.9)	1.59 (1.54–1.65)
No	Ref.	Ref.

Compared to those living in urban areas, coccidioidomycosis-associated hospitalisation was associated with residence in a large rural area (aOR 2.28 (95% CI 2.19–2.39)) and inversely associated with isolated rural areas (aOR 0.95 (95% CI 0.84–0.91))). Compared to those residing in higher (fourth quartile) income areas, coccidioidomycosis-associated hospitalisation was lower in those residing in first quartile (aOR 0.87 (95% CI 0.84–0.91)), second quartile (aOR 0.78 (95% CI 0.74–0.81)) and third quartile areas (aOR 0.87 (95% CI 0.83–0.81)). Coccidioidomycosis-associated hospitalisation was associated with Arizona residence (referent group: California) (aOR 6.42 (95% CI 6.25–6.60)).

Higher Elixhauser score was associated with coccidioidomycosis-associated hospitalisation (aOR = 1.02 (95% CI 1.02–1.03)) for each point in the Elixhauser score). Persons with AIDS and obesity had reduced odds of coccidioidomycosis-associated hospitalisation (aOR 0.75 (95% CI 0.58–0.99) and aOR 0.78 (95% CI 0.74–0.83), respectively). Uncomplicated diabetes (aOR 1.47 (95% CI 1.41–1.52)) and chronic pulmonary disease (aOR 1.59 (95% CI 1.54–1.65)) showed an increase in the odds of coccidioidomycosis-associated hospitalisation. Coccidioidomycosis-associated hospitalisation was associated with an increased mortality compared to the referent group who were alive at hospital discharge. (referent group: non-coccidioidomycosis-associated hospitalisation) (aOR 1.12 (95% CI 1.03–1.22)).

## Discussion

Using very large HCUP administrative datasets we had several novel observations related to coccidioidomycosis-associated hospitalisation, including relative incidence, association with important clinical, demographic and climatic factors, specifically rainfall. To the best of our knowledge, this is the first study to evaluate sociodemographic and comorbidity predictors of hospitalisations associated with coccidioidomycosis using a large inpatient population-based dataset.

We found many sociodemographic predictors such as gender, age, race/ethnicity and place of residence that had relationships with coccidioidomycosis-associated hospitalisation. Not surprisingly, African Americans had the highest odds of coccidioidomycosis-associated hospitalisation of any racial/ethnic group. African-American race is associated with a higher risk of disseminated infection and greater disseminated disease's severity [35]; thus the association with coccidioidomycosis-associated hospitalisation (compared to Caucasians) was expected. Using a California inpatient dataset, Sondermeyer *et al*. similarly found an increased relative risk for hospitalisation among African Americans consistent with our findings [21]. We found an increase in the odds of coccidioidomycosis-associated hospitalisation among persons in the age group 40–49 years and a lower odds for elderly patients. The increase for this age group may be attributed to the occupation. An occupational hazard for coccidioidomycosis infection has been shown among construction and agricultural workers who may belong to this age group [29]. Elderly patients may be hospitalised for other age-related conditions such as cardiovascular disease, malignancy and injury due to falls.

Interestingly, we found that lower income patient household (first through third quartile) had a lower odds for coccidioidomycosis-associated hospitalisation. This finding surprised us and the reasons for this association are unclear. It is possible that higher-income populations have better insurance and thus might have their coccidioidomycosis infection managed as an outpatient with a primary care provider, or perhaps are diagnosed earlier, preventing the need for coccidioidomycosis-associated hospitalisation.

Hospitalised patients in Arizona were at a much higher risk for coccidioidomycosis-associated hospitalisation than patients residing in California. Our findings are consistent with overall coccidioidomycosis incidence (hospitalised and non-hospitalised); a 2016 study found that Arizona accounted for 66% of cases while California accounted for 31% [20]. Of concern, Arizona has one of the fastest-growing populations in the United States, [36] which suggests that coccidioidomycosis-associated hospitalisation is likely to further increase in cases due to this influx of potentially susceptible persons. Our data suggest that not only are those residing in Arizona at higher risk for infection but at much higher risk for coccidioidomycosis-associated hospitalisation compared to Californians.

We also found that several comorbidities that were associated with coccidioidomycosis-associated hospitalisation, including uncomplicated diabetes and chronic obstructive pulmonary disease. Supporting our findings, a prior study showed diabetes to be a contributing cause of death in coccidioidomycosis patients, [37] suggesting diabetes may be associated with more severe coccidioidomycosis infection and coccidioidomycosis-associated hospitalisation. Additionally, patients with AIDS were at a lower risk for coccidioidomycosis compared to patients without AIDS. A study coinciding with the time frame of this study suggests a decrease in the incidence and severity of coccidioidomycosis within the HIV-1 infected population may be attributed to the introduction of potent antiretroviral therapy [38]. Our observation of lower risk of coccidioidomycosis-associated hospitalisation may be because persons with HIV are seen frequently by primary care practitioners for management of their HIV given their very high rates of insurance from Ryan White funding [39]. If persons with HIV or AIDS had coccidioidomycosis, they may have been diagnosed earlier, which precluded more serious illness and need for hospitalisation.

Interestingly, in our multivariable model, obese patients were at reduced risk of coccidioidomycosis-associated hospitalisation, which was an unexpected and novel finding. Possible reasons for this relationship are not readily apparent. Perhaps obese patients who were hospitalised were more likely to be hospitalised due to complications of obesity, such as cardiovascular disease. Higher comorbidity score was associated with coccidioidomycosis-associated hospitalisation (aOR = 1.02 (95% CI 1.02–1.03)) for each point in the Elixhauser score), suggesting that more severe forms of coccidioidomycosis requiring hospitalisation are more common in those with underlying co-morbidities. Studies examining coccidioidomycosis-associated death, which is obviously another severe complication of coccidioidomycosis, found several comorbidities associated with death [40]. Our study is the first study to include all 29 comorbid conditions present in the Elixhauser comorbidity index score. Although we did not exclusively look at fungal meningitis per se, a recent study showed that fungal meningitis due to either cryptococcus, coccidioidomycosis, histoplasmosis or candidiasis was also associated with more comorbid conditions [15]. Together these data suggest that higher comorbidities are associated with more severe coccidioidomycosis infection requiring hospitalisation.

The association between climate and coccidioidomycosis incidence is based on the ‘grow’ and ‘blow’ hypotheses [31]. This hypothesis argues that *Coccidioides* arthrospores are present at a higher concentration in the soil after heavy rains and as the soil dries up wind or soil disruption spreads the arthrospores [41, 42]. Interestingly, we found no correlations between coccidioidomycosis hospitalisations and seasonal precipitation levels for Arizona and paradoxically negative, albeit weak, correlations between coccidioidomycosis hospitalisations and seasonal precipitation levels for California. Our results support the more recent hypothesis that additional environmental factors such as wind, temperature and human activities play a more influential role than precipitation alone in explaining relationships between climate and incidence of coccidioidomycosis infections [43]. Alternatively, it could be that coccidioidomycosis-associated hospitalisation incidence follows a different pattern than overall coccidioidomycosis infection incidence and hence we are describing previously undescribed unique patterns.

Our study had limitations. First, our case definition included patients with a primary or secondary diagnosis code for coccidioidomycosis. Inclusion of a secondary diagnosis could result in an overestimation for the incidence of disease due to diagnostic coding error since the hospitalisation may have been due to a non-coccidioidomycosis primary diagnosis and the patient may have had chronic coccidioidomycosis that did not trigger the hospitalisation. However, among cases included based on a secondary diagnosis of coccidioidomycosis the two most prevalent primary diagnoses were HIV and pneumonia. Since HIV infection without complications is not a reason for hospitalisation and pneumonia could be related to coccidioidomycosis pulmonary infection, these patients' hospitalisations may be due to coccidioidomycosis. Additionally, our investigation followed previously reported methods for epidemiological study inclusion criteria [21]. Second, administrative databases are prone to missing or misclassifying diseases and comorbidities. Thus, selection bias may have been introduced, although it is difficult to know which direction any bias may have tilted our findings. Additionally, a limitation of comorbidities is that coccidioides may be underdiagnosed or patients have less aggressive investigations if milder cases, or those with intact immune systems, which may skew the incidence to patients with comorbidities. Future studies should evaluate the accuracy of billing coding for coccidioidomycosis. Third, we examined only inpatients for our investigation. Many severe infections may have been treated as outpatients and we could not measure the burden of hospitalisation compared to overall disease. The outpatient population was excluded from our study because information on outpatients is not collected in the SID. Fourth, our study population did not include those hospitalised in Indian Health Services facilities or federal hospitals, such as veterans. Thus, our population was not truly representative of all hospitalised patients in California and Arizona given we missed these populations. Finally, our comparison group is those who are hospitalised without coccidioidomycosis. Thus, we cannot estimate risks of coccidioidomycosis hospitalisation among the general population. Instead, our population was that of hospitalised patients and we found characteristics that were significantly high or low among those hospitalised with and without coccidioidomycosis.

Our investigation has strengths. First, ours is the first study to examine the association between coccidioidomycosis-associated hospitalisation and comorbidity measures including the relatively new Elixhauser index. Secondly, to our knowledge, our study is novel in that it utilised a large population-based cohort consisting of hospitalised patients from two highly endemic areas, Arizona and California where prior studies used both inpatient or outpatient cohorts from individual endemic states. Finally, we examined a large period of time from multiple SID databases, thus avoiding relationships that may be present only in low or high incidence years and the peculiarities specific to more epidemic years.

In conclusion, we found multiple sociodemographic predictors and comorbidities associated with coccidioidomycosis-associated hospitalisation in an inpatient dataset consisting of individual-level data obtained from the State Inpatient Datasets for Arizona and California. Disseminated coccidioidomycosis confers a high mortality rate and significant cost (an estimated $55 000 per hospital visit [44]). Therefore, early identification of populations at highest risk for coccidioidomycosis-associated hospitalisation may be helpful for limiting disease-associated morbidity, mortality and cost. Our results contribute to a better understanding of the inpatient coccidioidomycosis population. Studies like ours bring attention to healthcare provides of the potential disease burden attributed to coccidioidomycosis. Future research may wish to consider improved methods of early diagnosis and treatment modalities in high-risk populations to prevent coccidioidomycosis-associated hospitalisation.

## Conflict of interest

None.

## Data availability statement

The data that support the findings of this study are available from HCUP. Restrictions apply to the availability of these data, which were used under agreement for this study. Data are available from Deborah Kupferwasser with the permission of the HCUP.

## References

[1] Deresinski S (2019) Coccidioidomycosis: what a long strange trip it's been. Medical Mycology 57(Supplemental 1), 3–15. 10.1093/mmy/myy123 PMC6347081 30690606

[2] Anstead GM (2006) Coccidioidomycosis. Infectious Disease Clinics of North America 20, 621–643. 16984872 10.1016/j.idc.2006.06.005

[3] Brown J (2013) Coccidioidomycosis: epidemiology. Clinical Epidemiology 25, 185–197. 10.2147/CLEP.S34434 PMC3702223 23843703

[4] Lee L (2017) Increased coccidioidomycosis among inmates at a California prison. Journal of Correctional Health Care 23, 347–352. 28656821 10.1177/1078345817716451 PMC6951238

[5] Wilken J (2015) Coccidioidomycosis among workers constructing solar power farms, California, USA, 2011–2014. Emerging Infectious Diseases 21, 1997–2005. 26484688 10.3201/eid2111.150129 PMC4622237

[6] Lauer A (2020) Valley fever: environmental risk factors and exposure pathways deduced from field measurements in California. International Journal of Environmental Research and Public Health 17, 5285. 10.3390/ijerph17155285 PMC7432779 32707996

[7] Williams V (2018) Coccidioidomycosis, active component, U.S. armed forces, 2007–2017. Medical Surveillance Monthly Report 25, 2–5. 29696982

[8] Tsang C (2013) Increase in reported coccidioidomycosis – United States, 1998 to 2011. Morbidity and Mortality Weekly Report 62, 217–221. 23535687 PMC4605002

[9] Nelson R (2019) Valley fever on the rise after years of decline in the USA. The Lancet Infectious Diseases 19, 1173. 31657781 10.1016/S1473-3099(19)30576-6

[10] National Notifiable Diseases Surveillance System (NNDSS). (n.d.). Available at https://wwwn.cdc.gov/nndss/.

[11] Komatsu K (2003) Increase in coccidioidomycosis – Arizona. Morbidity and Mortality Weekly Report 52, 109–112. 12645841

[12] Vugia D (2009) Increase in coccidioidomycosis – California. Morbidity and Mortality Weekly Report 58, 105–109. 19214158

[13] Sondermeyer G (2017) Notes from the field: increase in coccidioidomycosis – California, 2016. Morbidity and Mortality Weekly Report 66, 833–834. 28796756 10.15585/mmwr.mm6631a4 PMC5687785

[14] Hector R (2011) The public health impact of coccidioidomycosis in Arizona and California. International Journal of Environmental Research and Public Health 8, 1150–1173. 21695034 10.3390/ijerph8041150 PMC3118883

[15] Charalambous L (2018) Prevalence, healthcare resource utilization and overall burden of fungal meningitis in the United States. Journal of Medical Microbiology 67, 215–227. 29244019 10.1099/jmm.0.000656 PMC6557145

[16] Saubolle M (2006) Epidemiologic, clinical, and diagnostic aspects of coccidioidomycosis. Journal of Clinical Microbiology 45, 26–30. 17108067 10.1128/JCM.02230-06 PMC1828958

[17] Galgiani J (2016) Infectious Diseases Society of America (IDSA) clinical practice guideline for the treatment of coccidioidomycosis. Clinical Infectious Diseases 63, 717–722. 27559032 10.1093/cid/ciw538

[18] Girois S (2006) Adverse effects of antifungal therapies in invasive fungal infections: review and meta-analysis. European Journal of Clinical Microbiology and Infectious Diseases 25, 138–149. 16622909 10.1007/s10096-005-0080-0

[19] Chu J (2006) Hospitalizations for endemic mycoses: a population-based national study. Clinical Infectious Diseases 42, 822–825. 16477560 10.1086/500405

[20] Luo R (2016) Hospitalized burden and outcomes of coccidioidomycosis: a nationwide analysis, 2005–2012. Medical Mycology 55, 368–374. 10.1093/mmy/myw087 PMC5896874 27703017

[21] Sondermeyer G (2013) Coccidioidomycosis-associated hospitalizations, California, USA, 2000–2011. Emerging Infectious Diseases 19, 1590–1597. 24050438 10.3201/eid1910.130427 PMC3810749

[22] US Census Bureau Public Information Office. 2007–2011 American Community Survey 5-Year Estimate – News Conferences – Newsroom – U.S. Census Bureau [Internet]. 2007–2011 American Community Survey 5-Year Estimate. Available at https://www.census.gov/newsroom/releases/archives/news_conferences/20121203_acs5yr.html.

[23] United States Department of Agriculture Economic Research Service. 2010 Rural-Urban Commuting Area Codes (RUCA) Report: Available at https://www.ers.usda.gov/data-products/rural-urban-commuting-area-codes/documentation/.

[24] Elixhauser Comorbidity Software, Version 3.7, Available at www.hcup-us.ahrq.gov/toolssoftware/comorbidity/comorbidity.jsp.

[25] Healthcare Cost and Utilization Project facts and figures, 2006: Statistics on Hospital-Based care in the United States, Agency for Healthcare Research and Quality. 21595103

[26] Moore B (2017) Identifying increased risk of readmission and in-hospital mortality using hospital administrative data. Medical Care 55, 698–705. 28498196 10.1097/MLR.0000000000000735

[27] Talamantes J (2007) Fluctuations in climate and incidence of coccidioidomycosis in Kern County, California: a review. Annals of the New York Academy of Sciences 1111, 73–82. 17347336 10.1196/annals.1406.028

[28] Schneider E (1997) A coccidioidomycosis outbreak following the Northridge, Calif, earthquake. The Journal of the American Medical Association 277, 904–908. 9062329

[29] Das R (2012) Occupational coccidioidomycosis in California. Journal of Occupational and Environmental Medicine 54, 564–571. 22504958 10.1097/JOM.0b013e3182480556

[30] Comrie C (2005) Climate factors influencing coccidioidomycosis seasonality and outbreaks. Environmental Health Perspectives 113, 688–692. 15929890 10.1289/ehp.7786 PMC1257592

[31] Drips W (1964) Epidemiology of coccidioidomycosis. The Journal of the American Medical Association 190, 1010–1012. 14214515

[32] National Oceanic and Atmospheric Administration. National Oceanic and Atmospheric Administration, Available at www.noaa.gov/.

[33] Kleinbaum D (2014) Applied Regression Analysis and Other Multivariable Methods. 5th Edn. Stamford, CT: Cengage Learning.

[34] Analytics, Business Intelligence and Data Management. Analytics, Business Intelligence and Data Management SAS, Available at www.sas.com/.

[35] Ruddy B (2011) Coccidioidomycosis in African Americans. Mayo Clinic Proceedings 86, 63–69. 21193657 10.4065/mcp.2010.0423 PMC3012635

[36] U.S. Census Bureau. National Population Totals and Components of Change Report: 2010–2019.

[37] Santelli A (2006) Coccidioidomycosis in patients with diabetes mellitus. The American Journal of Medicine 119, 964–969. 17071165 10.1016/j.amjmed.2006.03.033

[38] Masannat F (2010) Coccidioidomycosis in patients with HIV-1 infection in the era of potent antiretroviral therapy. Clinical Infectious Diseases; 50, 1–7. 19995218 10.1086/648719

[39] Health Resources & Services Administration: Ryan White HIV/AIDS Program. (2020) Available at https://hab.hrsa.gov/about-ryan-white-hivaids-program/who-was-ryan-white.

[40] Rosenstein N (2001) Risk factors for severe pulmonary and disseminated coccidioidomycosis: Kern County, California, 1995–1996. Clinical Infectious Diseases 32, 708–714. 11229838 10.1086/319203

[41] Jones J (2017) Coccidioidomycosis: an underreported cause of death – Arizona, 2008–2013. Medical Mycology 56, 172–179. 10.1093/mmy/myx041 PMC6080208 28595294

[42] Tamerius J (2011) Coccidioidomycosis incidence in Arizona predicted by seasonal precipitation. PLoS One 6, e21009–e21015. 21701590 10.1371/journal.pone.0021009 PMC3118810

[43] Weaver E (2018) Investigating the relationship between climate and valley fever (coccidioidomycosis). EcoHealth 15, 840–852. 30284073 10.1007/s10393-018-1375-9

[44] Freedman M (2018) Coccidioidomycosis outbreaks, United States and worldwide, 1940–2015. Emerging Infectious Diseases 24, 417–423. 29460741 10.3201/eid2403.170623 PMC5823332

